# Oligomerization of the heteromeric *γ-*aminobutyric acid receptor GABA_B_ in a eukaryotic cell-free system

**DOI:** 10.1038/s41598-022-24885-0

**Published:** 2022-12-01

**Authors:** Jessica Ullrich, Philip Jonas Göhmann, Anne Zemella, Stefan Kubick

**Affiliations:** 1 Fraunhofer Institute for Cell Therapy and Immunology (IZI), Branch Bioanalytics and Bioprocesses (IZI-BB), Am Mühlenberg 13, 14476 Potsdam, Germany; 2 Institute of Biotechnology, Technische Universität Berlin, Straße Des 17. Juni 135, 10623 Berlin, Germany; 3 Institute of Chemistry and Biochemistry-Biochemistry, Freie Universität Berlin, 14195 Berlin, Germany; 4 Faculty of Health Science, Joint Faculty of the Brandenburg University of Technology Cottbus-Senftenberg, the Brandenburg Medical School Theodor Fontane and the University of Potsdam, Potsdam, Germany

**Keywords:** Biochemistry, Biotechnology

## Abstract

Understanding the assembly mechanism and function of membrane proteins is a fundamental problem in biochemical research. Among the membrane proteins, G protein-coupled receptors (GPCRs) represent the largest class in the human body and have long been considered to function as monomers. Nowadays, the oligomeric assembly of GPCRs is widely accepted, although the functional importance and therapeutic intervention remain largely unexplored. This is partly due to difficulties in the heterologous production of membrane proteins. Cell-free protein synthesis (CFPS) with its endogenous endoplasmic reticulum-derived structures has proven as a technique to address this issue. In this study, we investigate for the first time the conceptual CFPS of a heteromeric GPCR, the *γ*-aminobutyric acid receptor type B (GABA_B_), from its protomers BR1 and BR2 using a eukaryotic cell-free lysate. Using a fluorescence-based proximity ligation assay, we provide evidence for colocalization and thus suggesting heterodimerization. We prove the heterodimeric assembly by a bioluminescence resonance energy transfer saturation assay providing the manufacturability of a heterodimeric GPCR by CFPS. Additionally, we show the binding of a fluorescent orthosteric antagonist, demonstrating the feasibility of combining the CFPS of GPCRs with pharmacological applications. These results provide a simple and powerful experimental platform for the synthesis of heteromeric GPCRs and open new perspectives for the modelling of protein–protein interactions. Accordingly, the presented technology enables the targeting of protein assemblies as a new interface for pharmacological intervention in disease-relevant dimers.

## Introduction

G protein-coupled receptors (GCPRs) represent the largest class of membrane proteins in the human organism. With over 800 members they fulfil a variety of essential physiological functions and play an important role in drug development and the pharmaceutical industry^1^. GPCRs have long been considered monomeric, but nowadays the formation of dimers and even higher order oligomers is widely accepted, while their functional significance and role in disease are still controversial^2^. Within the different classes of GPCRs (A–E), class C is a well-studied group partly requiring oligomeric assembly for functionality, such as for the metabotropic *γ*-aminobutyric acid receptor type B (GABA_B_)^3^. GABA_B_ is found pre- and postsynaptically and plays an essential role in various signal transduction pathways induced by its ligand *y*-aminobutyric acid (GABA), for example by influencing downstream effectors and modulating neuronal excitability as well as receptor-mediated signalling pathways^4^. As GABA_B_ plays a central role in neurobiology and is widely distributed, up-and downregulation of the receptor has been linked to a variety of neurological and psychiatric disorders such as schizophrenia, depression, drug addiction and neuropathic pain^5^. Despite extensive efforts in pharmacological research to modulate GABA-like ligands, only a few agonists, antagonists and allosteric modulators have been described to date. However as several drug candidates showed promising initial results, but were abandoned due to severe side effects, there is a growing demand for ligands and the development of novel pharmacological screening methods^6^. Biochemically, GABA_B_ forms an obligate heterodimer of two distinct protomers (BR1, BR2) interacting through strong non-covalent bonds^7^. More recently, dynamic self-assembly of heterodimeric subunits into higher order oligomeric assemblies has been reported^8,9^.

Structural analysis of GABA_B_ revealed, in addition to the characteristic seven transmembrane domains, an extracellular Venus flytrap domain (VFT) and a cytoplasmic tail for each subunit^10^. In vivo BR1 is retained in the endoplasmic reticulum (ER), unless the ER-retention motif is masked by a coiled-coil interaction with BR2, allowing transport to the plasma membrane and thus functionality^3^. In the assembled state, BR1 binds GABA in its VFT, and BR2 is involved in G- protein activation^7^, while homomers are reported inactive^11,12^.

However, the identification and in vitro study of membrane protein complexes with its functional properties and their pharmacological intervention remains challenging due to low protein yields in heterologous expression systems, difficulty in the stoichiometric alignment of interacting proteins, time-consuming and disrupting extraction procedures and the need for a suitable membrane environment for protein assembly and study^13–15^. Recently CFPS has emerged as a promising and efficient technology for the rapid synthesis of various classes of proteins in a well-defined environment, including GPCRs^16,17^, glycoproteins^18,19^ and oligomeric proteins^20,21^. Additionally, in previous studies, we reported CFPS of various monomeric GPCRs using lysates from eukaryotic insect cells originating from *Spodoptera frugiperda* (*Sf*21)^22^.

Accordingly, various efforts have been made over the last two decades to map protein assembly and protein–protein interactions in combination with CFPS, ranging from co-immunoprecipitation to fluorescence-based methods^23–30^. However, heteromeric GPCRs synthesized in a cell-free system have not been reported to our knowledge, although this would represent a significant extension of the proteomic toolbox.

In this study, we characterize the eukaryotic CFPS of a heteromeric GPCR, GABA_B_. In a first step, we validate a robust CFPS method, followed by a PLA to detect colocalization in endogenous microsomes and a bioluminescence energy resonance transfer (BRET) assay. The PLA and BRET showed spatial proximity of cosynthesized proteins, indicating GABA_B_ assembly in a eukaryotic CFPS system. These results provide a simple and powerful experimental platform for the CFPS of heteromeric GPCRs and point to further use in future interactome and pharmacological studies of small molecules targeting disease-relevant dimers.

## Results

### Establishing cell-free synthesis of GABA_B_

**Figure 1:**
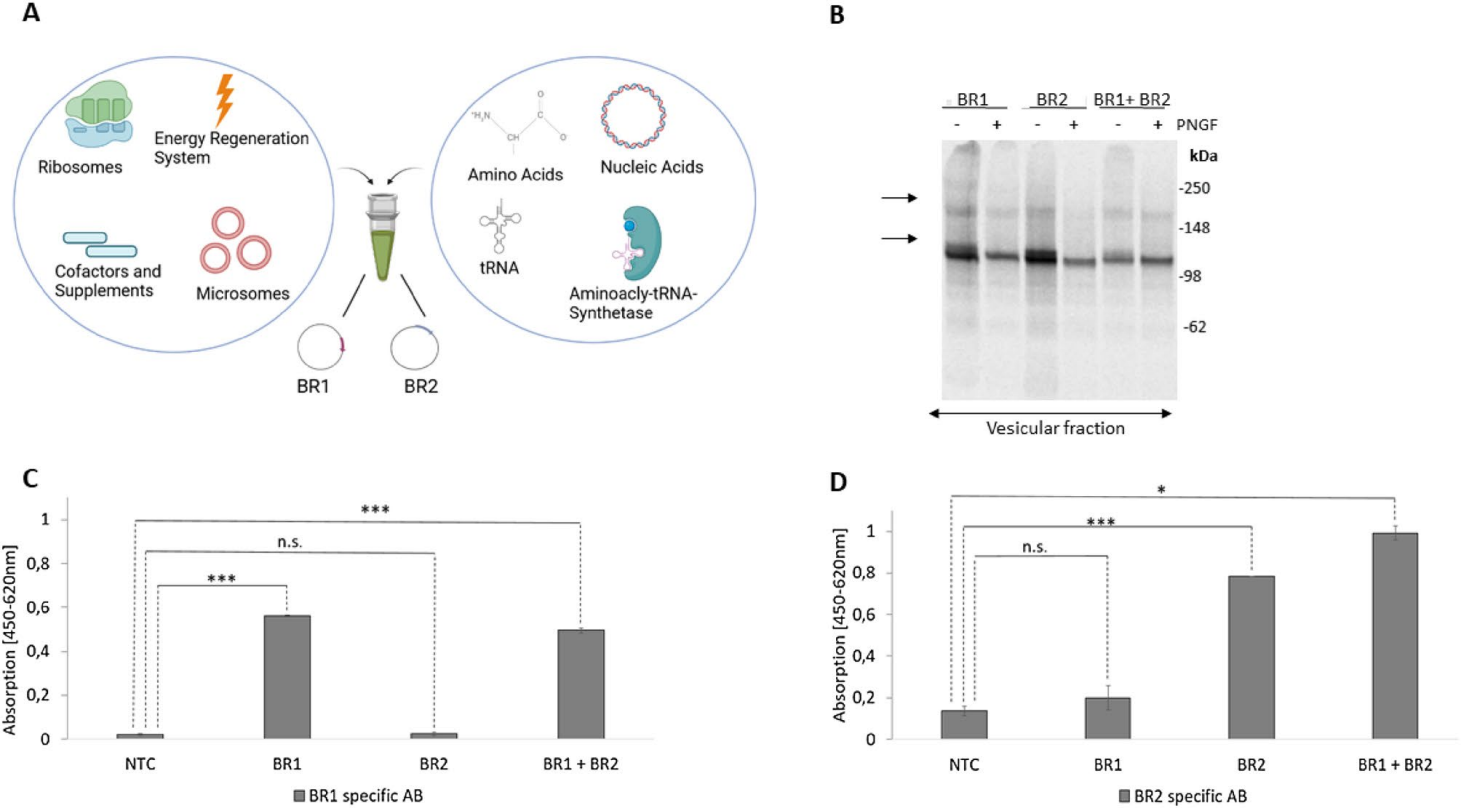
Cell-free protein synthesis of individually and coexpressed GABA_B_ forming subunits: (**A**) Schematic Illustration of cell-free synthesis of the GPCR, GABA_B_, in a eukaryotic cell-free system (*Sf*21). (**B**) Autoradiograph showing ^14^C-labeled individual cell-free synthesis and equimolar coexpression of subunits 1 (BR1) and 2 (BR2). Samples were PNGase F (PNGF) treated (plus and minus) and arrows indicate the corresponding bands (BR1: 109.7 kDa, BR2: 107.2 kDa). (**C**) Detection of cell-free synthesized proteins with rabbit primary antibody targeting BR1 subunit by an equimolar enzyme-linked immunosorbent assay (ELISA). Individual and coexpression of BR1 and BR2 were applied (n = 3). (**D**) Detection of cell-free synthesized proteins using primary mouse antibodies targeting BR2 by an equimolar ELISA (n = 3). Individual and coexpression of BR1 and BR2 were applied (n = 3). A p-value of < 0.05 was considered statistically significant and indicated by *, while a p-value < 0.001 is indicated by ***.

Although GPCRs are of considerable biological and pharmacological interest, many protein–protein interactions remain to be elucidated. In particular, the effects of complex assembly on receptor structure, dynamics and their implications for human pathology and physiology remain partially elucidated. CFPS, due to its open nature, allows flexible and controllable protein synthesis conditions based on the addition of any number or combination of DNA-encoding template plasmids to a translationally active lysate (Fig. 1a). In addition, microsomes harbouring active, cell-free synthesized membrane proteins can be directly applied to analytical procedures without prior purification steps as well as labeled with radioactively amino acids^22^. However, the feasibility of forming a GPCR heterooligomer using CFPS has not yet been demonstrated. Therefore, the synthesis of GABA_B,_ an obligatory heterooligomer of subunit 1 (BR1) and subunit 2 (BR2), is being examined in an insect based *Sf2*1 cell-free system.

Since for the heterologous synthesis of protein complexes, the most difficult subunit to synthesize is assembly regulating, both separate and simultaneous CFPS was assessed. Equimolar synthesis of the individual and coexpressed GABA_B_ subunits resulted in a comparable total protein yield of 10 µg/mL (Supplementary Fig. 1a). The protein yield is consistent with previous results for other GPCRs synthesized using CFPS and indicates similarly good manufacturability of both synthesized subunits^31^. The addition of ^14^C-leucine subsequently enabled the synthesized proteins to be analyzed autographically and revealed a broad monomeric band and a weak dimeric band (~ 13% of the total coexpressed protein, data not shown) after individual and coexpression of the subunits (Fig. 1b, Supplementary Fig. 1b) corresponding to the calculated molecular weights (BR1: 109.7 kDa, BR2: 107.2 kDa). However, the dimeric bands indicate the formation of homo- or heterodimers and require further investigation. Therefore, BR2 was fused with a fluorescent mCitrine (BR2-mCit, 134 kDa) and BR1 was fused to a nanoluciferase (BR1-NLuc, 128.7 kDa). The fusions constructs delivered also a monomeric and a band at higher molecular weight corresponding to the fused fluorescent proteins (Supplementary Fig. 1c). Since this shift could not be conclusively assigned to the homo- or heterodimer, we additionally coexpressed a fusion construct with the wildtype. This revealed a dimeric band with a slightly lower molecular weight than when the fusion constructs were co-expressed but slightly higher as the wildtype expression. This molecular weight shift in the dimeric band additionally suggests heterodimer formation.

The width of the monomeric bands was further investigated using a deglycosylation assay (PNGase F) (Fig. 1b), resulting in the partial disappearance of the monomeric band, thereby indicating the presence of *N*-glycosylations (BR1: 7*N*-glycosylations, BR2: 5*N*-glycosylations) and thus translocation of the subunits into the ER membrane. The endopeptidase PNGase F cleaves N-linked glycans between protein core asparagine and the *N*-acetylamine, resulting in a more homogenous, reduced mass and thus narrower band. This translocation is a mandatory prerequisite for the assembly of several membrane protein complexes and thus their functionality.

Additionally, an equimolar enzyme-linked immunosorbent assay (ELISA) was performed targeting the individual GABA_B_ subunits (Fig. 1c,d). Here, a sequence-specific antibody targeting BR1 showed a significant signal for both individual BR1 expression and coexpression. Similar data were found for an antibody directed against BR2, which showed a significant signal for the individual BR2 expression, as well as for coexpression. These results demonstrate the successful CFPS of the translocated, glycosylated GABA_B_ protomers.

### Proximity ligation assay indicates heterodimer formation

**Figure 2:**
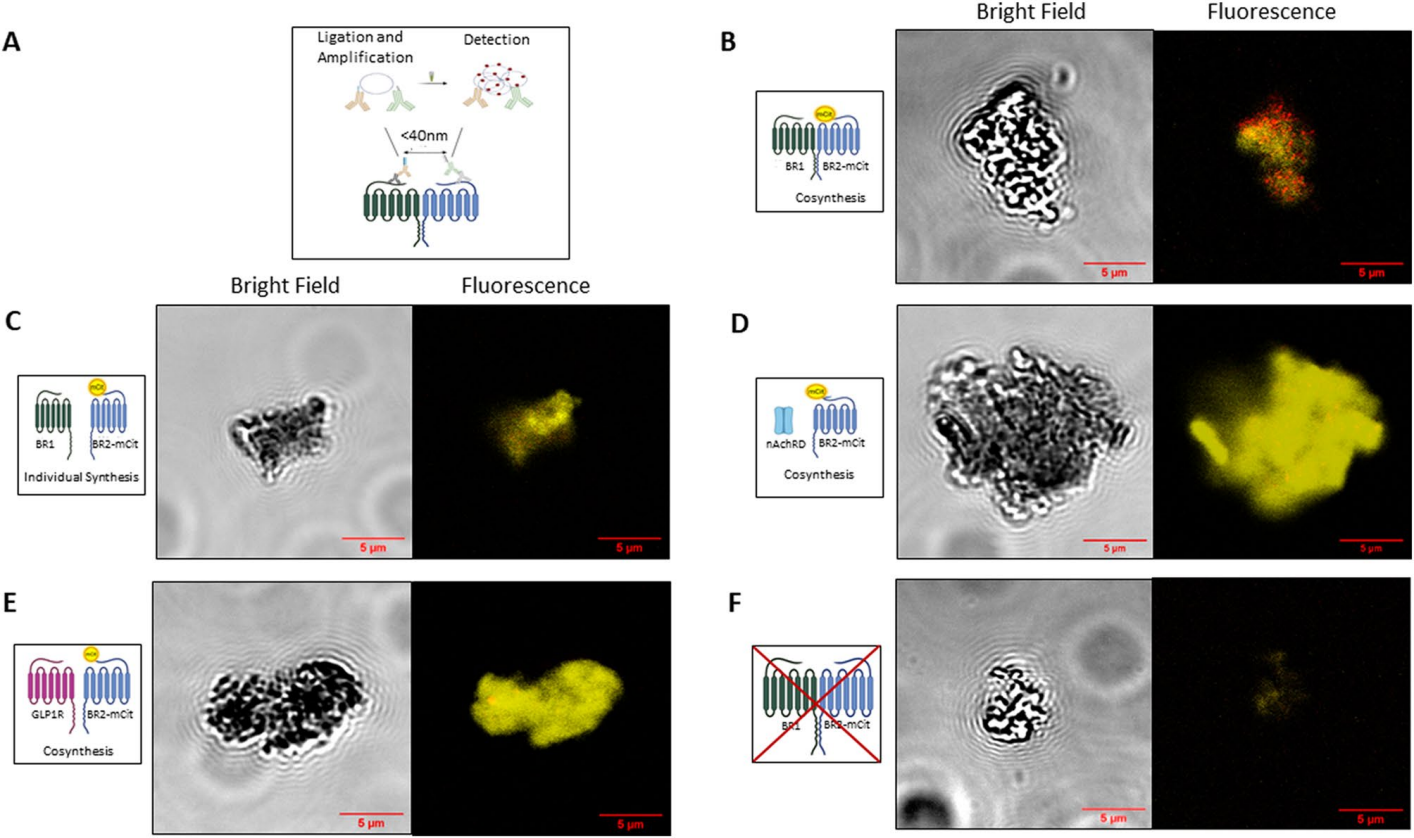
Detection of colocalized GABA_B_ by a proximity ligation assay (PLA): Samples were incubated with primary antibodies and secondary probes, ligated and amplified. The red fluorescent PLA signal corresponds to spatial proximity, while BR2-mCit alone emits yellow fluorescence. Analysis was performed by a confocal laser scanning microscope. (**A**) Conceptualization of PLA using Merck Duolink PLA Kit. (**B**) Representative coexpression of BR1 and BR2 to detect heterodimeric assembly. Primary antibodies were previously validated (Fig. 1d,e). (**C**) Negative control: Separate CFPS of BR1 and BR2-mCit translocated into different microsomes. Primary antibodies were targeting BR2 and the fused His-tag of the nicotinic acetylcholine receptor subunit delta (nAChRd). (**D**) Negative control: Coexpression of nAchRd with BR2-mCit. Primary antibodies were targeting the nAchRd His-Tag and BR2-mCit. (**E**) Negative Control: Coexpression of glucagon-like peptide 1 receptor (GLP1R) with BR2-mCit. Primary antibodies were directed against the GLP1R His-Tag and BR2-mCit. (**F**) Negative control: CFPS approach without coding DNA-templates. Primary antibodies were previously validated (Fig. 1d,e).

To investigate potential protein assembly of the GABA_B_ subunits into a receptor complex, a Duolink proximity ligation assay (PLA; Merck)was performed. This assay is based on the spatial proximity of two primary antibodies (< 40 nm) targeting interacting proteins with single-molecule resolution^32^. The previously validated primary antibodies were derived from different species and detected by secondary probes directed against the host organism. The spatial proximity subsequently enables ligation and PCR amplification of DNA strands conjugated to the secondary probes. Fluorescent DNA-binding probes are visualized as red dots by confocal scanning laser microscopy (CLSM), thereby indicating spatial proximity of the proteins of interest (Fig. 2a).

An important criterion for studying protein oligomerization is the colocalization of proteins within the same compartment. To investigate this for the cell-free synthesized proteins within the ER-derived microsomal structures, a PLA was applied. CLSM data of coexpressed subunits revealed bright fluorescence colocalized with a red PLA signal within the microsomes (Fig. 2b). Yellow fluorescence of the BR2-mCit is rather prominent compared to the isolated red dots of the heterodimers, since the quantification delivered an oligomerization rate of only 13%. A similar pattern was detectable for the coexpression of the fusion constructs BR1-NLuc and BR2-mCit (Supplementary Fig. 2b).

To control for the specificity of the PLA signal, the subunits were synthesized separately ensuring localization in different microsomes and were mixed together after synthesis. As expected, yellow fluorescence of the single BR2-mCit expression (Supplementary Fig. 2a) and coexpression was detectable, but no PLA signal (Fig. 2c). Similarly, no PLA signal was detectable when non-interacting proteins such as the delta subunit of the nicotinic acetylcholine receptor subunit delta (nAchRd) (Fig. 2d) and other GPCRs, the glucagon-like peptide 1 receptor (GLP1R) (Fig. 2e) and the gastric inhibitory polypeptide receptor (GIPR) (Supplementary Fig. 2c), were coexpressed with the BR2-mCit subunit. The negative control without coding plasmid showed a weak background fluorescence (Fig. 2f), while the single expression of BR2-mCit showed a strong yellow fluorescence. However, no PLA signal was detectable for either of them, while the positive control with the same primary antibody combination gave a strong red signal (Supplementary Fig. 2d).

Overall, these results demonstrate the spatial proximity of the subunits within the microsomes and indicate a heterodimeric arrangement of the protomers.

### BRET assay reveals GABA_B_ heterooligomer

**Figure 3:**
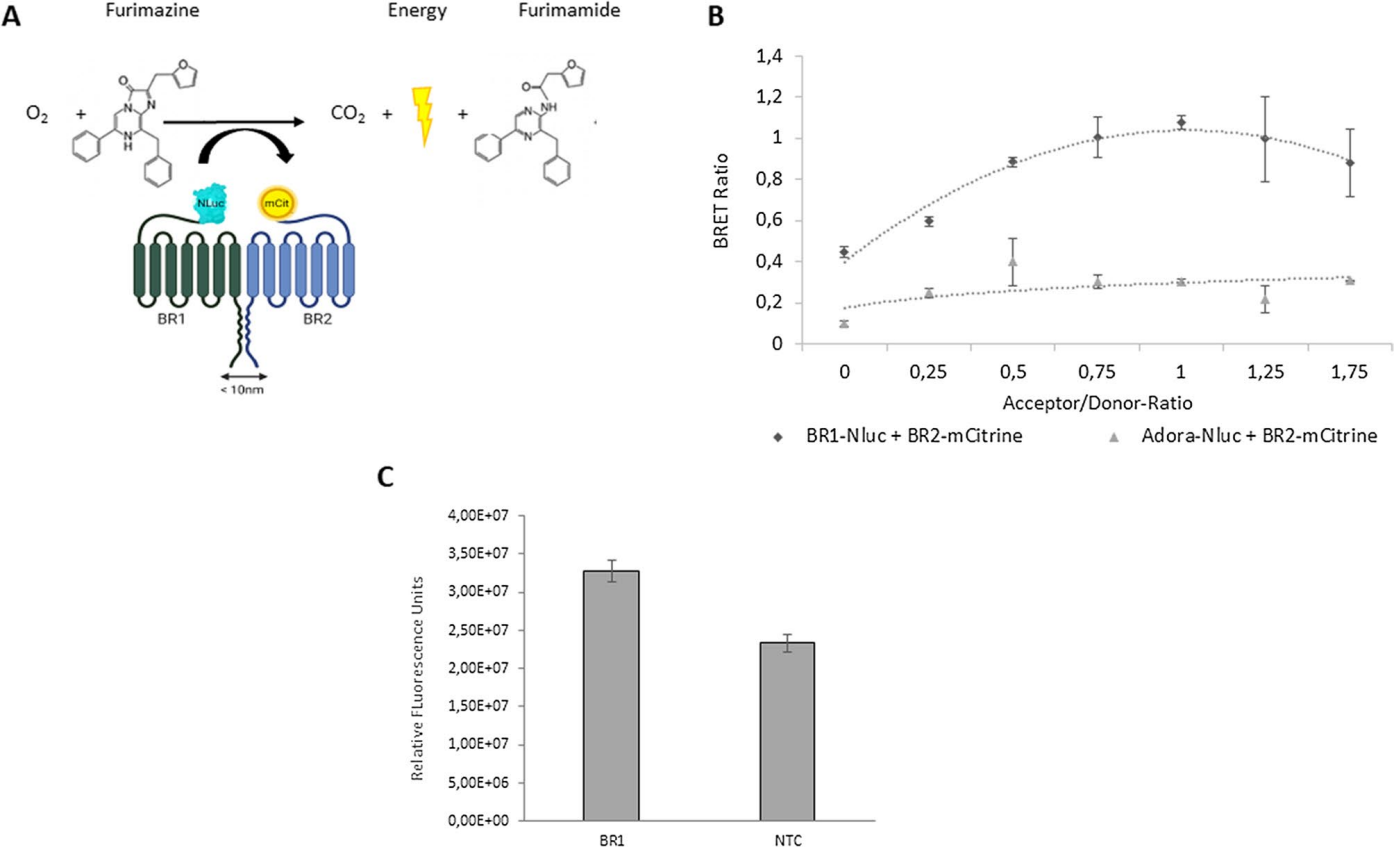
Bioluminescence Resonance Energy Transfer and antagonist binding: (**A**) Conceptualization of the performed BRET experiment: Coexpression of BR1-NLuc and BR2-mCit. If proteins interact, substrate oxidation results in excitation of BR2-mCit by non-radiative energy transfer. (**B**) Saturation BRET Experiment: Coexpression of BR1-NLuc and BR2-mCit with increasing donor concentration. Negative control: Adora-Nluc coexpressed with BR2-mCit. BRET ratio was calculated from triplicates (n = 3). (**C**) Binding of fluorescent antagonist (CPG 54626A) to BR1. Fluorescence was detected and quantified. Standard deviations were calculated from the triplicate analysis.

The gold standard for determining protein assembly are resonance energy transfer (RET) techniques such as the bioluminescence resonance energy transfer assay (BRET). To address this purpose, BR1-NLuc was synthesized using CFPS and screened for activity (Supplementary Fig. 3a). The addition of a luciferase substrate (furimazine) oxidizes the nanoluciferase and the energy is transferred to the fluorescent BR2-mCit. Once donor and acceptor molecules are below 10 nm apart, the energy is transferred via nonradiative dipole–dipole interaction, resulting in a light signal corresponding to the emission of the acceptor (Fig. 3a)^33^. Saturation assays are most prevalent for studying oligomerization of GPCRs^34^, especially if oligomerization is not externally inducible. Therefore, a BRET saturation assay was performed with a fixed amount of BR1-NLuc coding plasmid coexpressed with increasing BR2-mCit plasmid concentrations. Coexpression of the labeled constructs showed fluorescence, whereas CFPS of the unlabeled protein showed no fluorescence signal (Supplementary Fig. 3b-d). This setup was transferred to the BRET assay, where coexpression of BR2-mCit with the adeno-A2A receptor fused to a nanoluciferase (Adora-NLuc) resulted in a linear BRET ratio with increasing plasmid concentration. This negative control excluded the possibility that the BRET signal resulted only from colocalization of the proteins within the same microsomes or overexpression. Instead, the BRET ratio curve of coexpressed BR1-NLuc and BR2-mCit showed a hyperbolic shape with a saturation level. These results indicate that the donor molecules of BR1 interact with the proximate BR2 molecules and thus form a heterooligomer. They confirm the previous PLA results and suggest a heterodimeric arrangement of the GABA_B_ subunits in a eukaryotic CFPS system based on *Sf*21 cells.

To further demonstrate suitability of the synthesis and pharmacological screening of a heterodimeric GPCR, we performed a fluorescence based antagonist binding study (Fig. 3c). The orthosteric antagonist (CGP 54626A) binds to the target protein specifically in its VFT region^3^. A strong fluorescence signal for the antagonist binding to the sample is detectable, while negative control without coding DNA plasmid, showed a much weaker fluorescence signal. The overall high background of the experiment is due to the lipophilic character of the antagonist, which appears to interact slightly with the microsomal membrane. The suitability of cell-free synthesized heterodimeric GPCRs and their use for pharmacological applications have been demonstrated accordingly.

## Discussion

GPCRs play critical roles in many processes in the human body and are the most prominent drug target^1^. Due to their nowadays widely accepted oligomerization, the knowledge of monomeric GPCRs as a functional entity has been turned upside down, and the alternated mechanisms, functionality, and role of oligomers in health need to be viewed and questioned in a detached manner^2^. This opens new perspectives on functional protein complexes, their medical role and intervention^35^. However, the lack of knowledge regarding protein–protein interactions and oligomerization of some GPCR is partially linked to challenges in heterologous expression and the requirement of a suitable surrounding environment to introduce protein assembly. Heterologous GPCR expression is commonly performed in mammalian cells due to the complex nature of these proteins with regards to folding and stability. However, the need for stoichiometric synthesis conditions, cytotoxicity, receptor internalizations, as well as perturbing isolation procedures for functional and pharmacological screenings are challenging^13–15,36,37^.Multiple quality control points and chaperones are required for proper protein quality^38^, which are difficult to control in cell-based approaches due to the background of endogenous components. Therefore, due to its open nature, CFPS offers defined and customizable synthesis conditions allowing the addition of radioactive substances, cytotoxic components as well as pharmaceutically relevant components such as ligands and antagonists. Furthermore, the defined addition of DNA-coding plasmids based on their stoichiometric composition enables rapid control of protein synthesis in coexpression and mutation studies within a few hours without DNA truncation and exposure to perturbing detergents. Using CFPS, BR1 can be analyzed independently of BR2. In conventional cell-based experiments, BR1 requires assembly with BR2 for transport to the plasma membrane and the associated accessibility for various assays. Using CFPS, either isolated or simultaneous coexpressed analysis without purification in a natural like environment is possible.

Accordingly, we conceptually demonstrate the CFPS of the well-studied GABA_B_ in a eukaryotic cell-free system as a flexible technique to synthesize heteromeric GPCRs. This receptor is a *bona fide* GPCR model protein and has been reported to form heteromeric assemblies in hippocampal neurons and various eukaryotic cell lines in heterologous overexpression^7,9–12^. We validate the CFPS of the coexpressed subunits of obligate heteromeric GABA_B_ by the addition of ^14^C-leucine with a corresponding autoradiography and an immunoassay. Oligomerization of GABA_B_ was found to be induced by interactions of a salt bridge as well as hydrophobic and hydrogen bonds formed by VFT, while the oligomer is stabilized by c-terminal coiled-coil interactions^39,40^. Accordingly, low and high molecular weight bands corresponding to homodimers and heterodimers are found in the autoradiograph at the underlying SDS-PAGE, as previously described in literature^41^. Further autoradiographic validation by coexpression with fusion constructs indicated the correspondence of the dimeric band to a heterodimer (Supplementary Fig. 1c).

Since many GPCRs require glycosylations and an appropriate membrane environment for protein functionality and assembly^42^, we detected *N*-glycosylations using a deglycosylation assay demonstrating translocation of synthesized proteins into ER-derived microsomes, which is an essential prerequisite for post-translational modifications (Fig. 1b). Given the pharmacological relevance of GPCRs, the glycosylation structures are of particular importance for potential therapeutic and screening applications^43^, as well as for protein assembly. The narrowing of the autoradiographic bands after the deglycosylation assay is consistent with previous reports, where 7 glycosylation sites are described for BR1 and 5 for BR2. In previous studies, we demonstrated the ability of the CFPS system to synthesize core *N*-glycosylations^18^. Nevertheless for upcoming functional CFPS based screenings of GPCRs, the demonstrated lower heterogeneity of the sugar moieties have to be considered carefully.

Additionally, we combined PLA and CFPS to demonstrate the localization of the proteins within the microsomal structures as well as colocalization (Fig. 2b). Due to the open nature of CFPS, the potential proximity of the subunits was modulated by synthesizing the proteins in different microsomes hindering dimerization since the subunits appear to be in a range of 1–1.5 µm apart from each other^44^ (Fig. 2c). Generally, PLA allows visualization of protein proximity with a high degree of spatial accuracy (< 40 nm) and shows a high sensitivity comparable to a standard polymerase chain reaction^32,45,46^. In literature, colocalization of receptor heteromers is defined as the essential basis of receptor oligomerization^47^. Compared to other methods, this assay is universally applicable without extensive cloning procedures. Microscale thermophoresis and surface plasmon resonance-based assays are commonly used alternatives. However, these methods require a complex and cost-intensive instrumental setup. In addition, the use of surface plasmon resonance analysis is partly limited by the immobilization of the proteins, thereby influencing protein stability and affecting potential binding sites. Accordingly, using oligomer-disrupting ligands for screening applications is only possible to a limited extent. Instead, PLA can be performed with any pair of antibodies, allowing any codetection of proteins without extensive labelling, solubilisation or critical immobilization procedures that may interfere with functionality. It has been used previously for several GPCR studies to investigate heterodimerization, including the cannabinoid, dopamine and adenosine receptors in native tissue^48,49^ as well as for the GABA_B_ receptor^50,51^. However, in combination with eukaryotic CFPS this technique was applied for the first time and indicates a heterodimer formation of the proteins.

Resonance energy transfer (RET) techniques, including the bioluminescence (BRET) assay, have long been the gold standard to detect protein–protein interactions within < 10 nm^34,52^, but require elaborate cloning procedures. To further validate, an additional BRET saturation assay was performed using subunit fusion constructs resulting in a saturation curve (Fig. 3b). In cell-based studies, a corresponding result was previously found^53^. Accordingly, the BRET results reinforce the PLA finding and demonstrate a heterodimeric assembly of GABA_B_ in the CFPS system_._ As drugs targeting the interaction of GABA_B_ heterodimers have rarely been found, cell-free synthesized GABA_B_ could be used for initial screens targeting neurological and psychiatric disorders by antagonists, such as small peptides^35^ similar to the antinociceptive bivalent ligand targeting the heteromeric mGlu5-*µ*-opioid GPCR^54^.

The combination of heterodimeric GPCR synthesis using the CFPS system presents an intriguing addition to the proteomics toolbox. In a further application, it could be combined with previously described automated, chip-based CFPS systems, allowing broad pharmacological screenings^55,56^. For such, colocalization is a first step, but more detailed functional screenings have to be established. A typical downstream assay that, is based on the classical GTPγS Gα binding^57^. Here the coupling of G-proteins after ligand binding is analyzed. Therefore, the presence of Gα has to be verified in the cell-free system. Alternatively, all subunits of the Gα protein or mini G-proteins might be co-synthesized to the target protein or added exogenously. GTP binding and hydrolysis is afterwards monitored. A second approach to visualize downstream functionality is based on the binding of β-arrestin to the target protein after activation. Therefore, the C-terminus of the receptor and the β-arrestin are labeled with fluorescent tags such as YFP and GFP. After activation and β-arrestin binding, the fluorescent proteins are in close proximity and an energy transfer can be measured^58^. A prerequisite for this approach is the presence of G protein-coupled receptor kinase that phosphorylates the C-terminus of the GPCR upon activation. This has to be evaluated before performing experiments.

However, we demonstrated the binding of an orthosteric antagonist using a fluorescence assay confirming protein quality in the pharmacologically highly relevant VFT domain and the applicability of pharmaceutical screenings. A possible application of such binding screening of potential drug candidates in combination with functional assays could address questions of ligand binding and receptor oligomerization, for example in the controversially discussed family of class A and orphan GPCRs^59^. The transfer of the presented techniques to other protein–protein interactions would be conceivable. However, the studies were performed partially in the presence of solubilizing agents, which may have altered the antagonist binding properties of the protein^60^. Due to its open nature, CFPS additionally allows the synthesis of a versatile protein library with variable mutation, protein tags and targeting motifs, enabling now a flexible, facile screening for oligomerization. However, production of high protein yields and upscaling in CFPS are challenging. For screenings only low protein amounts are required, but once it is a matter of high-resolution structure determination, comparatively high protein yields have to be achieved by extensive upscaling.

These results reveal a facile and powerful experimental platform for the synthesis of heteromeric GPCRs, rising new perspectives for modelling protein–protein interactions, ligand bindings and mutational studies. Establishing this methodology will allow a better understanding of the different compositions of GPCR complexes and can be used as an interface for targeting protein compositions as pharmacological interventions in disease-relevant dimers.

## Materials and methods

### Batch-based cell-free synthesis

Translationally active lysate was prepared as previously described^61^. A coupled protein synthesis was performed in batch mode with a reaction made of 40% (v/v) *Sf*21 cell lysate, amino acids (complete 100 µM, Merck), Mg(OAc)_2_ (f.c. 3.9 mM, Merck), KOAc (f.c. 150 mM, Merck), spermidine (f.c. 0.25 mM, Roche), T7 RNA polymerase (1 U/mL, Invitrogen), UTP (0.3 mM, Roche), CTP (0.3 mM, Roche) and 0.1 mM of the cap analogue m7G(ppp)G. To provide energy, a regeneration system containing creatine phosphate (20 mM, Roche), creatine phosphokinase (f.c. 0.1 mg/mL), ATP (1.75 mM, Roche) and GTP (f.c. 0.3 mM, Roche) was added to the reaction. Furthermore, polyG primers (f.c. 10 µM, IBA) and radioactive ^14^C-leucine (f.c. 50 µM, specific radioactivity 66.7 dpm/pmol, Perkin Elmer) were used.

Plasmids were synthesized de novo (Biocat GmbH) based on sequence motifs published by Ref.^62^. The human GABA_B_ sequence (uniprot: BR2 O75899, BR1 Q9UBS5) was fused to an internal ribosome entry site (IRES) originating of the cricket paralysis virus (CrPV) and the native signal sequence was replaced by the honeybee melittin signal sequence, which enables a better translocation of the proteins into the microsomes^44^. Other proteins were designed accordingly; Nanoluciferase^63^ and mCitrine^64^ were fused N-terminally. Human Adora receptor (uniprot: P29274) was fused to a nanoluciferase^63^ as previously described by Ref.^65^. Nicotinic acetylcholine receptor subunit delta with a c-terminally 10xHis-tag (uniprot: Q07001). Gastric inhibitory protein receptor (uniprot: P09681) and glucagon-like 1 receptor (uniprot: P43220) with a c-terminally 10xHis-tag.

Plasmids were expressed either individually or equimolar simultaneously. In the negative control (NTC), the plasmid was replaced by water. Synthesis was performed in a thermocycler (Eppendorf SE) at 27 °C for 3 h at 600 rpm. After incubation, the translation mixture was centrifuged at 16,000*g*, 10 min, 4 °C, resulting in a supernatant and a vesicular fraction (VF). The latter contains the putative translocated membrane proteins and was therefore resuspended in phosphorus buffered saline (PBS; VWR) or sucrose (VWR).

### Quantitative analysis of synthesized proteins

Synthesized proteins were analyzed based on the integration of ^14^C-labeled leucine in the cell-free reaction and subsequent hot trichloroacetic acid (TCA; Carl Roth) precipitation. 3 µL sample were taken and consequently precipitated using 10%TCA (v/v) offset with 2% (v/v) casein hydrolysate (Carl Roth GmbH). The mixture was incubated for 15 min at 80 °C and cooled down for 30 min on ice. Using a vacuum filtration system non incorporated ^14^C leucine was removed by running the sample over a filter with a molecular weight cut-off of 10 kDa (MN GF3, Machery-Nagel). The filter unit was washed twice with 5% TCA and dried with acetone (Carl Roth GmbH). Afterwards, 3 mL of scintillation liquid (Quicksafe A, Zinsser analytic) was added to the filter and incubated for 1 h on an orbital shaker. The incorporated ^14^C-leucine was determined using liquid scintillation counting (Hidex 600SL, Hidex). The total protein content was calculated by the following equations based on the scintillation counts and protein specific parameters:

1 $$Protein\,Yield \left[\frac{\mu g}{mL}\right]=\frac{scintillation\,counts \left[\frac{dpm}{mL}\right]*molecular\,weight [\frac{\mu g}{pmol}]}{specific\,radioactivity \left[\frac{dpm}{pmol}\right]*number\,of\,leucines},$$

2 $$Specific\,radioactivity \left[\frac{dpm}{pmol}\right]=\frac{stock\,concentration\,of\,{}^{14}C\,Leucine\left[\mu M\right]*{A}_{spec}[\frac{dpm}{pmol}]}{total\,concentration\,of\,leucine [\mu M]}.$$

For coexpressed proteins, the total protein yield was estimated based on the sum of the molecular weights and the corresponding leucine concentration of the subunits. The analysis was performed in triplicates and the standard deviation was calculated.

### SDS-PAGE and autoradiography

The molecular size of the synthesized proteins was estimated using a SDS-PAGE and associated autoradiography. For this, 3 µL of the synthesized proteins were sampled and precipitated by the addition of ice-cold acetone (Carl Roth GmbH). After 15 min of incubation, samples were centrifuged at 16000*g*, 10 min, 4 °C to separate the precipitated protein and subsequently, air-dried. The protein pellet was resuspended in LDS sample buffer (ThermoFisher Scientific). After loading the sample on a precast Novex SDS-Gels (ThermoFisher Scientific), samples were run for 55 min. at 160 V at room temperature, stained (SimplyBlue Safe Stain, Life Technologies) dried at 70 °C on Whatman paper (Unigeldryer 3545D, Uniequip) and incubated in a phosphor screen for several days (GE Healthcare). The radiolabeled protein was visualized using a phosphor imager system (Amersham RGB Imager, GE Healthcare). The densitometric analysis of the complex formation was performed using ImageQuant TL software (TL 8.1- GE; Healthcare Bio-Science).

### Deglycosylation assay

A deglycosylation assay was performed using peptide *N*-glycosidase F (PNGase F, New England Biolabs GmbH) on ^14^C-labeled protein. The assay was performed according to the manufacturer’s instructions followed by SDS-PAGE and autoradiograph image analysis.

### Plate enzyme-linked immunosorbent assay

On a high binding plate (Corning), the synthesized protein was incubated overnight at 4 °C. On the next day supernatant was removed, the plate was washed three times with PBS-Tween (0.05%(v/v)) (VWR) and blocked with PBS-Tween and 2 mg/mL bovine serum albumin (Merck) for 2 h while shaking at 70 rpm. After another washing step, the primary antibodies were added (1:1000 in PBS, 1 mg/mL BSA): BR1: ABIN 486307 (Antibodies Online), BR2: 393270 (Santa Cruz Biotechnology). After 2 h incubation and washing, the secondary antibodies coupled to horseradish peroxidase were added for another 2 h: Rabbit-HRP (Santa Cruz Biotechnology), Mouse-HRP (Advansta Inc.). For detection, 50 µL of tetramethylbenzidine substrate (life technologies) was added and incubated until a blue staining was detectable. Finally, the reaction was stopped by adding 50 µL 0.5 M sulfuric acid. Samples were measured using an Omega Plate Reader (BMG Labtech), subtracting the emission at 450 nm from 640 nm. Samples were calculated from three independent experiments (n = 3). Kolomogorov-Smirnov-Test was performed. Additionally, a f-test and t-test for two independent samples was performed with p ≤ 0.05 (*) and p ≤ 0.001 (***).

### Fluorescence antagonist binding assay

VF of samples was solubilized. Samples were then centrifuged at 16,000*g*, 10 min, 4 °C and 10 nM linker (CGP 54626A (HelloBio)) was added to the supernatant. Using amicon ultra- 0.5 centrifugal filter unit (Merck, MWCO: 100 kDa) samples were diluted in PBS and passed through a filter unit at 14,000*g*, 15 min. Filters were flipped, samples evaluated at 3000*g* for 5 min and transferred to IBIDI 18-well μ-slides. Using a phosphor imager system (649 nm, Amersham RGB Imager, GE Healthcare) sample were analyzed and quantified using ImageQuant TL software (GE Healthcare). Analysis was performed in triplicates and standard deviation was calculated.

### Proximity ligation assay

Proximity ligation assay (PLA) was performed using the Duolink in situ Red Starter Kit Mouse/Rabbit” (Merck) with the following modifications: The protocol was adapted to IBIDI 18-well μ-slides (IBIDI GmbH) using a volume of 20 μL per sample and incubations steps were performed in a humidity chamber. Buffers from the plate ELISA described above were used until incubation with the secondary probes, for which buffers from the kit were applied. After synthesis, the VF was resuspended in 2.5 M sucrose (1:2 dilution) (VWR) and incubated overnight at 4 °C on the μ-slide. After removal of the supernatant, the samples were washed thrice with PBS-Tween (0.05%), blocked with 2 mg/mL BSA in PBS-T (0.05%) for 1.5 h and subsequently incubated with the primary antibodies (see above, additionally: His-Tag Antibody: MA1-21315, invitrogen) for 1.5 h, followed by washing with PLA Kit buffer A. Secondary probes were incubated with samples for 2 h. After another washing the ligase was added and incubated at 37 °C for 45 min. For amplification, samples were incubated for 100 min at 37 °C. Samples were washed twice with Kit washing Buffer B for 10 min and once with a 1:10 dilution of Washing Buffer B for 1 min. Duolink PLA mounting medium was added and samples were analyzed using a confocal laser microscope (LSM 510, Carl Zeiss Microscopy GmbH) with a 63×/1.4 oil objective. MCitrine was excited with an argon laser at 488 nm. After passing a long-pass filter (LP 505), the emitted light was captured using a photomultiplier. For PLA signal detection samples were excited with a HeNe laser at 543 nm. After passing a long-pass filter (LP 650), the emitted light was detected.

### Bioluminescence resonance energy transfer measurement

Saturation BRET experiments were performed constantly using 20 nM BR1-NLuc/Adora-NLuc and increasing BR2/BR2-mCitrine plasmid concentrations (0–50 nM) for CFPS. A 1:50 dilution of Nano-Glo^®^ luciferase substrate (Promega GmbH) to 5 µL of the VF was added. The assay was performed using a white plate in a Mithras^2^ LB 943 multimode reader (Berthold Technologies). Raw values were measured and calculated as emission intensity at 530 nm divided by emission intensity using the OD2 filter:

3 $${BRET}_{ratio}=\frac{Fluorescence\,(sample)}{Luminescence\,(sample)}-\frac{Fluorescence\, \left(control\right)}{Luminescence\, \left(control\right)}.$$

The BRET ratio was calculated as the ratio of a coexpressed sample with receptor and donor tag subtracted from the raw value obtained by coexpressing the Nluc-labelled receptor and the corresponding unlabelled receptor. The BRET ratio was calculated from three independent experiments (n = 3) and mean values and standard deviation were calculated and plotted.

The corresponding fluorescence detection was performed using 18-well μ-slides (IBIDI GmbH). Samples were transferred and at the excitation wavelength 488 nm using an Amersham RGB Imager (GE Healthcare).

## Supplementary Information

Supplementary Information.

## Data Availability

All relevant data are within the paper and its Supporting Information files.
