# Supplementary material for: Oligomerization of the heteromeric γ-aminobutyric acid receptor GABAB in a eukaryotic cell-free system
Source: Sci Rep. 2022 Dec 1;12:20742. doi: 10.1038/s41598-022-24885-0 (PMC9715706; doi:10.1038/s41598-022-24885-0)
Supplement: Supplementary file 1 — Supplementary Information. [file 41598_2022_24885_MOESM1_ESM.docx]

**Oligomerization of the heteromeric *γ-*Aminobutyric Acid Receptor GABA_B_ in a eukaryotic Cell-free System**

Jessica Ullrich^1,2^, Philip Jonas Göhmann^1,2^, Anne Zemella^1^, Stefan Kubick^1,3,4^

^1^ Fraunhofer Institute for Cell Therapy and Immunology (IZI), Branch Bioanalytics and Bioprocesses (IZI-BB), Am Mühlenberg 13, 14476 Potsdam, Germany

^2^ Technische Universität Berlin, Institute of Biotechnology, Straße des 17. Juni 135, 10623 Berlin, Germany

^3^ Freie Universität Berlin, Institute of Chemistry and Biochemistry- Biochemistry, 14195 Berlin, Germany

^4^ Faculty of Health Science, Joint Faculty of the Brandenburg University of Technology Cottbus- Senftenberg, the Brandenburg Medical School Theodor Fontane and the University of Potsdam, Germany

* Stefan.kubick@izi-bb.fraunhofer.de.

# Supplements


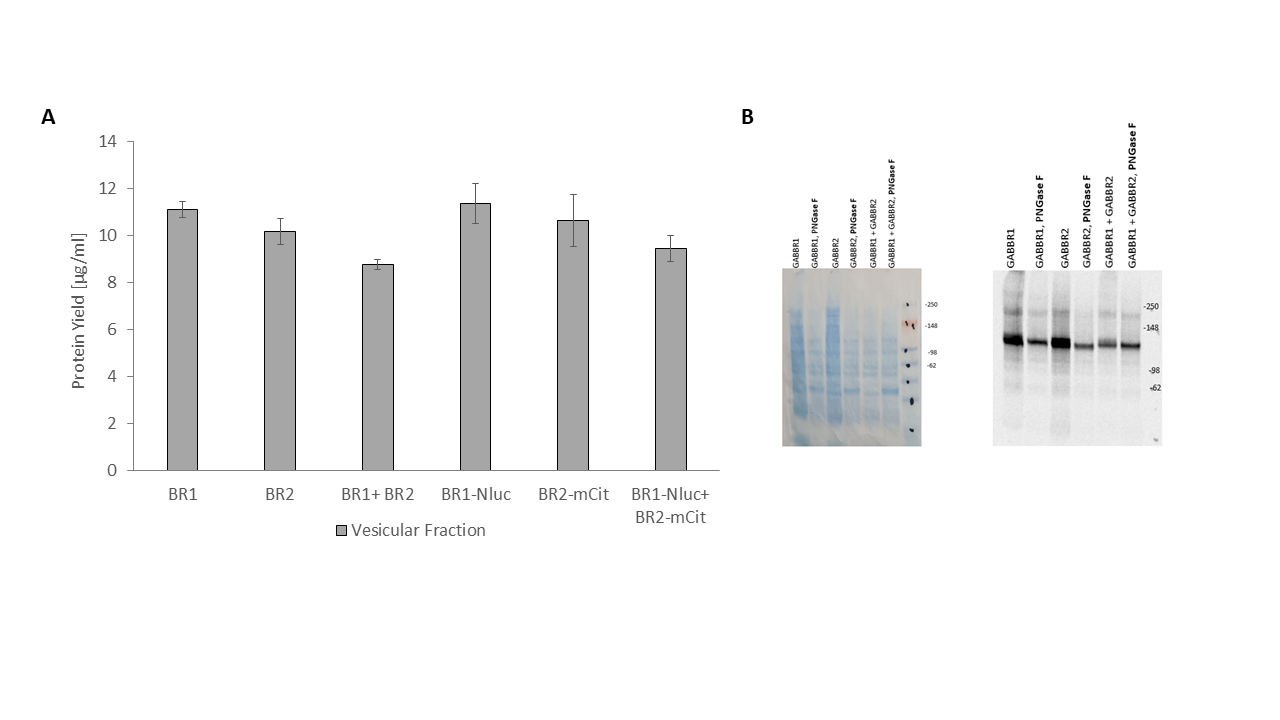


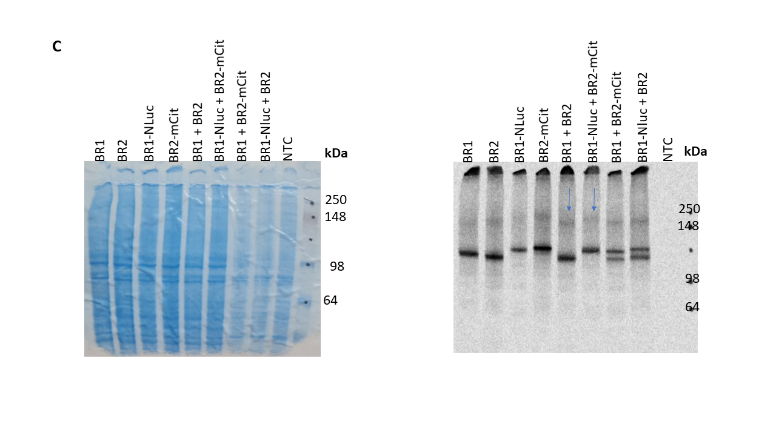


**Figure S 1 Cell-free synthesis of GABA_B_: (A)** Quantitative analysis of vesicular fraction by hot TCA-precipitation of ^14^C-leucine of individual or coexpressed labeled BR1 and BR2 detected by liquid scintillation counting. Additionally, fusion constructs of BR1-NLuc and BR2-mCit were analyzed. The total protein yield for simultaneous synthesis was estimated using the sum of the molecular weight and leucines of the expressed subunits. Standard deviations were calculated from the triplicate analysis. **(B)** Qualitative analysis of ^14^C-leucine labelled, coexpressed and glycodigested constructs of GABA_B_ by autoradiography Left: Coomassie stained SDS-PAGE, right: autoradiograph. **(C)** Qualitative analysis of ^14^C-leucine labelled, coexpressed fusion constructs of GABA_B_ by autoradiography. NTC: No template control, Left: Coomassie stained SDS-PAGE, right: autoradiograph.


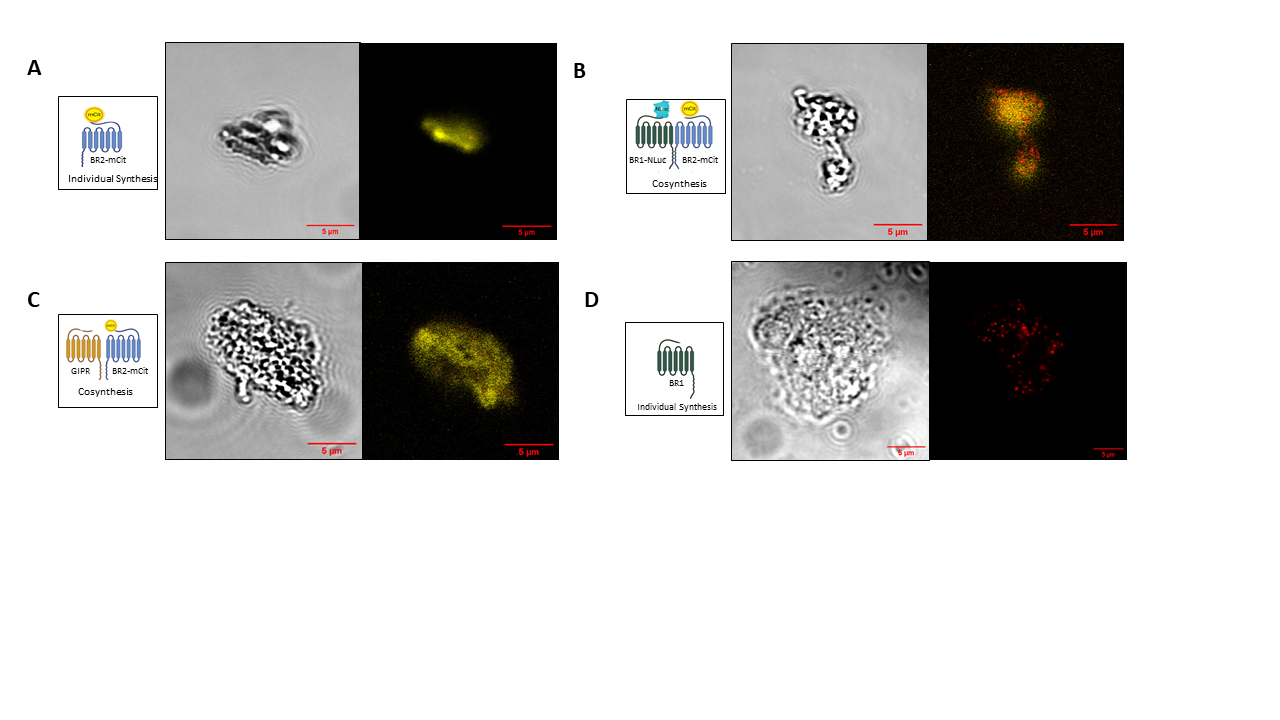


**Figure S 2 Further controls of Proximity Ligation Assay:** Samples were incubated with primary antibodies and secondary probes, ligated and amplified. The red fluorescent PLA signal corresponds to spatial proximity, while yellow fluorescence is caused by BR2-mCit. Analysis was performed by a confocal laser scanning microscope. Secondary probes were not varied. **(A)** Negative control: CFPS of BR2- mCit. Primary antibodies targeting BR1 and BR2 sequences were validated. **(B)** Coexpression of BR1-NLuc and BR2-mCit incubated with primary antibodies targeting either BR1 or BR2 sequences. **(C)** Negative Control: Coexpression of GIPR and BR2-mCit. Primary antibodies targeting either His-Tag of GIPR or BR2 sequence. **(D)** Positive Control: Individual Expression of BR1. Primary antibodies targeting either His-Tag or BR1 sequence.


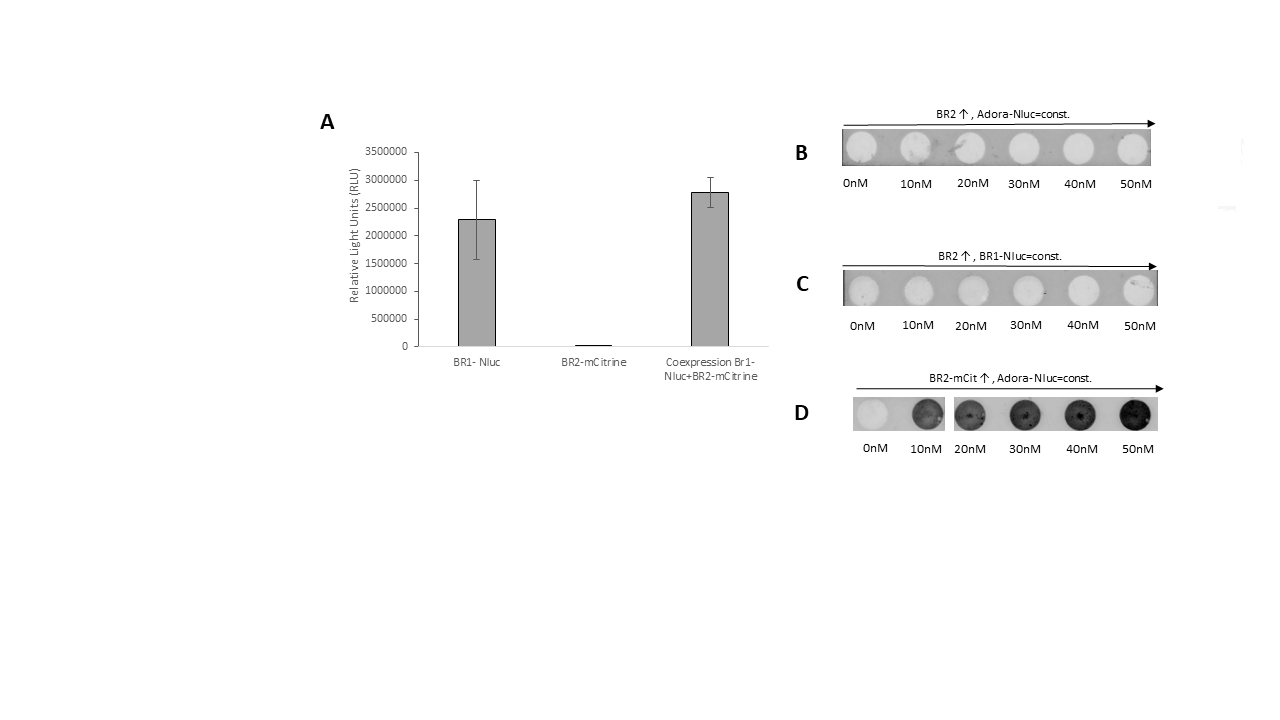


**Figure S 3 CFPS of GABA_B_ fusion constructs and their coexpression: (A)** Nanoluciferase activity of BR1-NLuc, BR2-mCit and coexpression. Measurements were performed as triplicates using Mithras^2^ LB 943 multimode reader. **(B)** Fluorescence analysis with increasing BR2 plasmid concentrations, while Adora-NLuc plasmid concentration is constant. **(C)** Fluorescence analysis of coexpression with increasing BR2 plasmid concentration, while BR1-NLuc is constant. **(D)** Fluorescence analysis of coexpression with increasing BR2-mCit plasmid concentration, while Adora-NLuc concentration is constant.

Fluorescence analysis of C-F was performed using Amersham RGB Imager.
